# Stigma among patients with TB infection in a low-TB-burden setting

**DOI:** 10.5588/ijtldopen.26.0070

**Published:** 2026-08-11

**Authors:** J.I. Campbell, A. Garing, D. Lavache, R. Quintero, S. Bahad, S. Goodwin, P. Sinha, T.C. Bouton, C. Acuna-Villaorduna, K. Traber, M. Canning, V. Sabharwal, K.R. Jacobson

**Affiliations:** 1 Section of Pediatric Infectious Disease, Boston Medical Center, Boston, MA, USA;; 2 Boston University Chobanian & Avedisian School of Medicine, Boston, MA, USA;; 3 Boston University School of Public Health, Boston, MA, USA;; 4 Section of Infectious Disease, Boston Medical Center, Boston, MA, USA;; 5 Department of Pulmonary, Allergy, Sleep, and Critical Care Medicine, Boston Medical Center, Boston, MA, USA;; 6 Boston Medical Center, Boston, MA, USA;; 7 Boston Public Health Commission, Boston, MA, USA.

**Keywords:** tuberculosis, TBI, TPT, language, genderstudy, and critically revised the manuscript

Dear Editor,

Diagnosing and treating TB infection (TBI) is a critical aspect of TB control.^1^ TB stigma and lack of social support are barriers to engagement in TB care,^2^ and recent qualitative data suggest that these factors may also contribute to gaps in care for TBI.^3^ However, the prevalence of stigmatising attitudes among individuals with TBI, differences between distinct populations seeking care, and the relationship between TB stigma and TBI care cascade outcomes have not been widely examined in low-TB-burden settings.^3,4^ A single prior survey of Haitian Creole–speaking patients with TBI in the USA characterised discordance between patients’ stigma experiences and clinicians’ perceptions of patient-level stigma.^5^

To inform stigma mitigation efforts in our setting, we conducted a prospective observational cohort study to quantify TB stigma among patients with TBI attending a hospital-based, public health TB clinic in Boston, USA, between August 2024 and June 2025. This project was nested within an evaluation of these patients’ contact networks. We recruited patients with a positive tuberculin skin test or interferon-gamma release assay who had been referred for evaluation and treatment of TBI. We included patients at initial clinical evaluations when treatment recommendations were made and at follow-up visits for patients receiving TB preventive treatment. When patients were <18 years old, we recruited their caregivers. We excluded patients who did not speak English, Spanish, or Haitian Creole (the predominant languages spoken by patients in the clinic), and those who were unhoused or who did not receive primary care within the clinic’s health organisation (requirements of the parent study).

We assessed TB stigma using the Van Rie TB stigma questionnaire,^6^ which queries individuals with TB about self, enacted, and anticipated stigma in two separate subscales: 1) personal perspectives about TB (‘individual stigma’) and 2) community perspectives about TB (‘community stigma’). Individual TB stigma was represented as a continuous variable from 0 (least stigma) to 36 (most stigma), and community TB stigma was represented as a continuous variable from 0 (least stigma) to 33 (most stigma). This questionnaire has been previously translated into Spanish.^7^ A certified medical interpreter translated the questionnaire into Haitian Creole, which was reviewed and back-translated independently by native-speaking research staff (Supplementary Data). We also elicited information about all members of the participant’s household and asked if the participant had told adult household members about his or her TBI diagnosis. We calculated whether the participant had disclosed to any adult household member, and also whether the individual had disclosed to the majority (>50%, high disclosure) or minority (≤50%, low disclosure) of adults within the household. We assessed TBI treatment completion by reviewing documented treatment completion in medical records. Patients were considered to not have completed therapy if they did not initiate treatment despite a recommendation or if they discontinued therapy before completing a standard duration of treatment. Patients who remained on treatment at time of the final study record review were considered to have completed treatment. Exposure variables were self-reported age, sex, language, and non-US birth. We used descriptive and summary statistics to analyse the cohort. To compare the degree of community versus individual TB stigma, we normalised each scale and compared means using paired *t* tests. We used multivariable linear and logistic regression to test associations between participant characteristics and TB stigma and treatment completion, using forward selection with a variable inclusion threshold of *P* < 0.2.

Fifty-three participants were included in the primary analysis (Supplementary Data Table S1). The median community stigma score was 18 (interquartile range [IQR] 13, 21) (possible range 0–33). The median individual stigma score was 18 (IQR 15, 23) (possible range 0–36). Participants had higher normalised mean community stigma scores than individual stigma scores (4.1% difference, *P* < 0.001). Among participants with adult household members with whom they could disclose their status (n = 48), 73% (n = 35) had disclosed to any adult household member and 67% (n = 32) had disclosed to more than 50% of adult household members. The distribution of stigma and disclosure by participant characteristic is shown in Supplementary Data Figure S1. In adjusted analysis (Supplementary Data Table S2), female participants trended toward higher community stigma scores than male participants (adjusted β 4.03 [95% confidence interval (CI): −0.03, 8.10]), and Spanish-speaking participants had lower community stigma scores than English-speaking participants (adjusted β −7.38 [95% CI: −13.59, −1.18]). Increasing age was associated with decreasing odds of disclosure to any adult household member (adjusted odds ratio [aOR] 0.93 per year [95% CI: 0.88, 0.99]). Compared to English-speaking participants, Haitian Creole–speaking participants had lower odds of disclosing to a high proportion (≥50%) of household members (aOR 0.11 [95% CI: 0.02, 0.60]). Overall, 61% of participants had completed preventive treatment. Stigma scores and disclosure were not significantly associated with treatment completion, although individuals who completed treatment had slightly higher community stigma scores (median 19 vs. 15, *P* = 0.264) – see Supplementary Data Figure S2.

In this cross-sectional analysis of TB stigma and disclosure among patients being evaluated and treated for TBI in a hospital-based, public health TB clinic, patients with TBI endorsed high rates of community and individual TB-related stigma, and moderate rates of disclosure within their households. Our findings contribute to insights about how TB-related stigma varies by demographic characteristics and between population groups. Studies from high-TB-burden settings have identified higher degrees of TB disease stigma among women compared to men.^8,9^ We found a trend towards increased stigma scores among women, likely reflecting previously observed patterns among individuals with TB disease. Our results suggest that methods to reduce TB stigma in our population may benefit from tailoring to different sexes. Likewise, we found decreased rates of stigmatising attitudes among participants who spoke Spanish compared to those who spoke English and Haitian Creole, and less disclosure among patients who spoke Haitian Creole. A prior review documented cultural variability in TB stigma,^10^ and our study suggests that this finding may extend to different cultural and language groups within the USA with TBI.

Within the context of patients receiving TB evaluation and care in a dedicated TB clinic, our findings add to an understanding of how stigma affects completion of TBI care. We found only a weak trend towards decreased treatment completion with increasing stigma, though our small sample size may have limited our ability to detect a significant relationship. Prior qualitative studies in individuals with TBI identified misperceptions about the difference between TB infection and disease leading to fear of contagion^11–13^; perception that TB is a ‘dirty’ disease that connotes poverty and thus leads patients to avoid testing and care to avoid these labels^11,12,14^; and perception that TB testing and care could lead to interaction with government authorities.^15^ These observations suggest that stigma may largely act upstream of patients reaching a TB clinic setting to start therapy – that is, in the cascades steps where most attrition occurs. Our findings lend qualified support to this hypothesis. Because our study population had already engaged in testing, and because participants were starting or had already started preventive treatment, understanding stigma attitudes in upstream steps of the TBI care cascade should be evaluated in future research.

Our study has several limitations. First, we only enrolled patients who had already reached our TB clinic, and our findings may not generalise to those individuals who exit the care cascade prior to clinic attendance. Second, our small sample size precluded well-powered multivariable analysis; our findings should be considered exploratory and merit corroboration in a larger study. Finally, several factors may limit generalisability to other settings and populations, including limited language coverage, exclusion of patients who were unhoused or who had not received primary care, and enrolment in a setting with state-supported TB care.

In conclusion, among patients being evaluated and treated for TBI in a public health-supported, hospital-based TB clinic in the USA, patients with TBI experienced high degrees of community and individual stigma. Stigma differed by sex and language, indicating that approaches tailored to specific population groups may be most effective to mitigate stigma in this setting. Future research is needed to characterise the relationship between stigma, disclosure, and TBI care prior to patients reaching TB clinics.

## ETHICAL STATEMENT

The Boston University Medical Campus IRB approved this study and all included patients provided written informed consent.

## Supplementary Material
