# Supplementary material for: Stigma among patients with TB infection in a low-TB-burden setting
Source: IJTLD Open. 2026 Aug 11;3(8):534–6. doi: 10.5588/ijtldopen.26.0070 (PMC13460433; doi:10.5588/ijtldopen.26.0070)
Supplement: Supplementary file 1 [file ijtldopen26-0070_supplementarydata1.pdf]

Supplemental Figure 1

Demographic characteristics, stigma, and disclosure

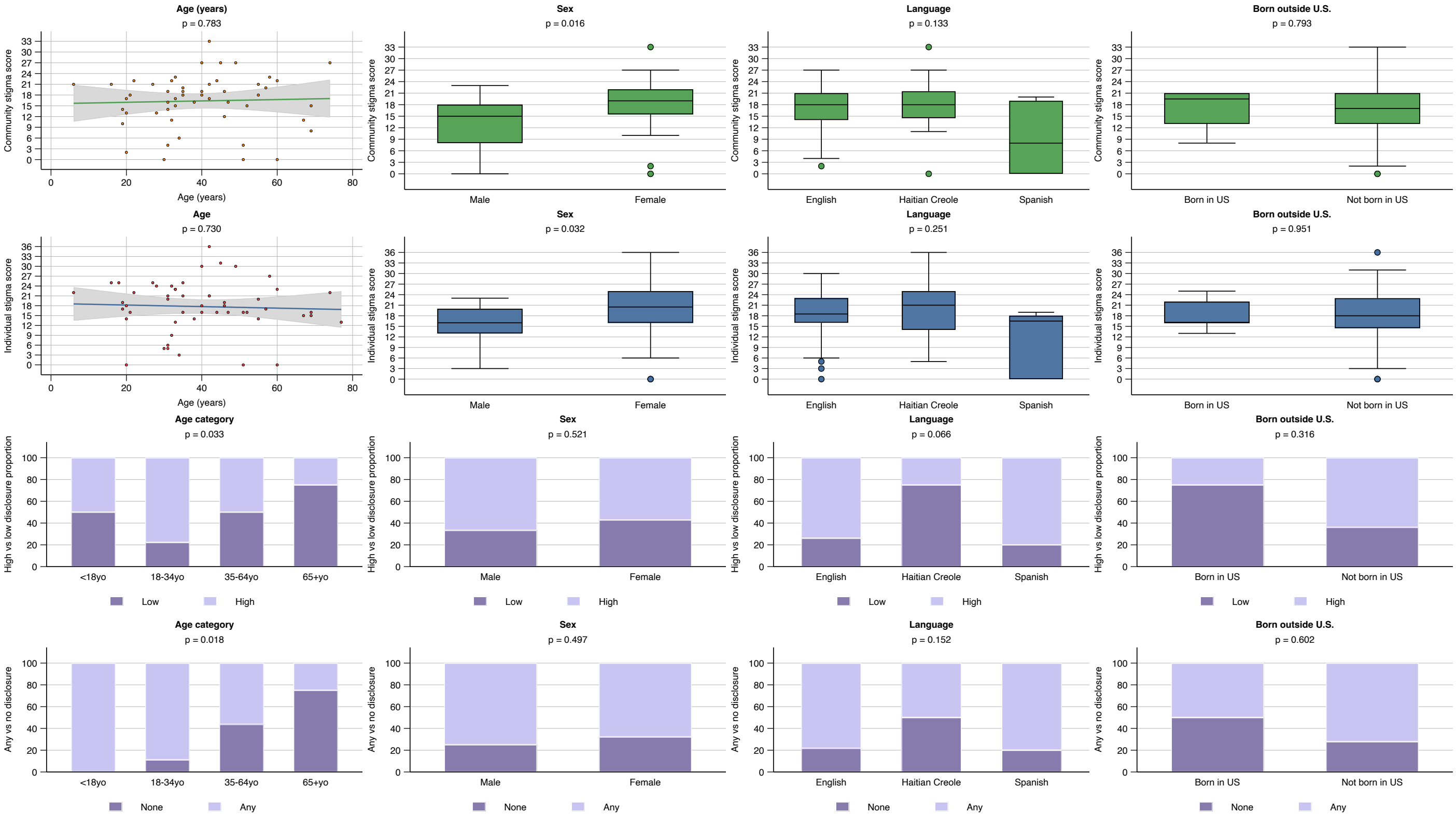

Supplemental Figure 2

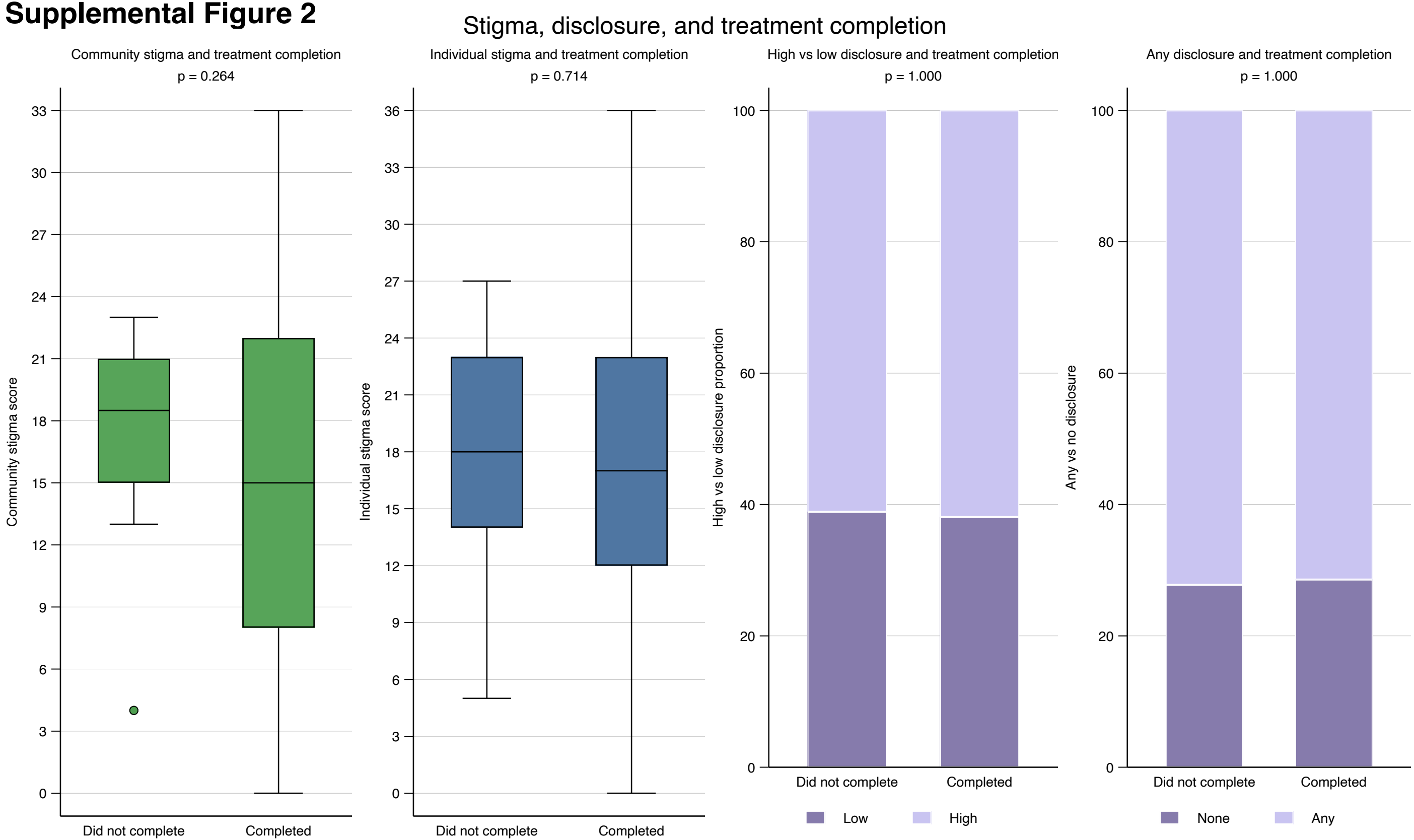

Supplemental table 1. Participant characteristics, stigma, and disclosure.

Characteristic	Participants N = 53	Median (IQR) community stigma score	Median (IQR) individual stigma score	Any disclosure (disclosure to any adult household members)	High disclosure (disclosure to >50% of adult household members)
**Age**	38 (31, 51)	NA	NA	NA	NA
**Age category**					
<18 years old	2 (4%)	21 (21, 21)	24 (22, 25)	2/2 (100%)	1/2 (50%)
18-34 years old	21 (40%)	15 (11, 19)	18 (9, 23)	19/21 (90%)	18/21 (86%)
35-64 years old	25 (47%)	19 (16, 22)	18 (16, 23)	13/21 (62%)	12/21 (57%)
65+ years old	5 (9%)	13 (10, 21)	15 (15, 16)	1/4 (25%)	1/4 (25%)
**Sex**					
Male	19 (36%)	15 (8, 18)	16 (13, 20)	13/16 (81%)	12/16 (75%)
Female	34 (64%)	19 (16, 22)	21 (16, 25)	22/32 (69%)	20/32 (63%)
**Language**					
English	30 (57%)	18 (14, 21)	19 (16, 23)	22/27 (81%)	21/27 (78%)
Haitian Creole	17 (32%)	18 (15, 22)	21 (14, 25)	9/16 (56%)	7/16 (44%)
Spanish	6 (11%)	8 (0, 19)	17 (0, 18)	4/5 (80%)	4/5 (80%)
**US birth**					
Yes	5 (9%)	20 (13, 21)	16 (16, 22)	3/5 (60%)	2/5 (40%)
No	48 (91%)	17 (13, 21)	18 (15, 23)	32/43 (74%)	30/43 (70%)
**Treatment completion***					
Completed treatment	30 (61%)	15 (8, 22)	17 (12, 23)	15/21 (71%)	13/21 (62%)
Did not complete treatment	19 (39%)	19 (15, 21)	18 (14, 23)	13/18 (72%)	11/18 (61%)

*Information available for 49 patients

Supplemental table 2. Regression analysis of characteristics associated with stigma and disclosure, using a forward-selection variable inclusion approach for multivariable models.

**Characteristic**	**Community stigma** (per 1-point increase in stigma score – corresponding to increased stigma)		**Individual stigma** (per 1-point increase in stigma score – corresponding to increased stigma)		**Any disclosure** (disclosure to any adult household members)		**High disclosure** (disclosure to >50% of adult household members)	
	Unadjusted β	Adjusted β	Unadjusted β	Adjusted β	Unadjusted OR	Adjusted OR	Unadjusted OR	Adjusted OR
**Age (per year)**	0.02 (-0.12, 0.16)	NA	-0.02 (-0.16, 0.11)	NA	0.95 (0.90, 0.99)	**0.93 (0.88, 0.99)**	0.96 (0.92, 1.00)	0.95 (0.90, 1.00)
**Sex**								
Male	Ref	Ref	Ref	Ref	Ref	NA	Ref	NA
Female	4.59 (0.42, 8.76)	4.03 (-0.03, 8.10)	3.97 (-0.45, 8.39)	3.25 (-1.15, 7.66)	0.51 (0.12, 2.19)	NA	0.56 (0.15, 2.12)	NA
**Language**								
English	Ref	Ref	Ref	Ref	Ref	Ref	Ref	Ref
Haitian Creole	1.17 (-3.26, 5.60)	0.51 (-3.85, 4.86)	2.39 (-2.25, 7.03)	1.86 (-2.79, 6.50)	0.29 (0.07, 1.17)	0.23 (0.04, 1.30)	**0.22 (0.06, 0.85)**	**0.11 (0.02, 0.60)**
Spanish	-7.73 (-14.11, -1.35)	**-7.38 (-13.59, -1.18)**	-6.00 (-12.83, 0.83)	-5.67 (-12.44, 1.09)	0.91 (0.08, 9.99)	2.55 (0.20, 32.61)	1.14 (0.11, 12.25)	1.30 (0.10, 17.68)
**US birth**								
Yes	Ref	NA	Ref	NA	Ref	NA	Ref	Ref
No	-0.70 (-8.57, 7.16)	NA	-0.71 (-8.19, 6.76)	NA	1.94 (0.29, 13.17)	NA	3.46 (0.52, 23.23)	11.65 (0.89, 152.37)

Supplemental table 3. Median and mean scores for individual items on the community and individual stigma scales.

Stigma scales	Median (IQR)*	Mean (SD)*
**Community stigma**		
A1. Some people may not want to eat or drink with friends who have TB	2 (1, 2)	1.70 (1.01)
A2. Some people feel uncomfortable about being near those with TB	2 (1, 2)	1.77 (0.85)
A3. If a person has TB, some community members will behave differently towards that person for the rest of his/her life	1 (1, 2)	1.28 (0.84)
A4. Some people do not want those with TB playing with their children	2 (1, 2)	1.81 (0.87)
A5. Some people keep their distance from people with TB	2 (2, 2)	1.81 (0.90)
A6. Some people think that those with TB are disgusting	1 (0, 2)	1.11 (0.91)
A7. Some people do not want to talk to others with TB	1 (1, 2)	1.24 (0.92)
A8. Some people are afraid of those with TB	2 (1, 2)	1.43 (0.93)
A9. Some people try not to touch others with TB	2 (1, 2)	1.41 (0.90)
A10. Some people may not want to eat or drink with relatives who have TB	2 (1, 2)	1.62 (0.90)
A11. Some people prefer not to have those with TB living in their community	1 (1, 2)	1.23 (0.85)
**Individual stigma**		
B1. Some people who have TB feel hurt because of how others react when they find out they have TB	2 (1, 2)	1.74 (0.92)
B2. Some people who have TB lose friends when they share with them they have TB	1 (1, 2)	1.34 (0.92)
B3. Some people who have TB feel alone	1 (1, 2)	1.43 (0.91)
B4. Some people who have TB keep their distance from others to avoid spreading TB germs	2 (2, 3)	1.98 (0.91)
B5. Some people who have TB are afraid to tell those outside their family that they have TB	2 (1, 2)	1.66 (0.98)
B6. Some people who have TB are afraid of going to TB clinics because other people may see them there	1 (0, 2)	1.13 (0.96)
B7. Some people who have TB are afraid to tell others that they have TB because others may think that they also have AIDS	1 (1, 2)	1.43 (0.99)
B8. Some people who have TB feel guilty because their family has the burden of caring for them	2 (1, 2)	1.45 (0.80)
B9. Some people who have TB will choose carefully who they tell about having TB	2 (1, 2)	1.83 (0.91)
B10. Some people who have TB feel guilty for getting TB because of their smoking, drinking, or other careless behaviors	1 (1, 2)	1.36 (0.90)
B11. Some people who have TB are worried about having AIDS	1 (1, 2)	1.08 (0.83)
B12. Some people who have TB are afraid to tell their family that they have TB	1 (1, 2)	1.32 (0.86)

*Possible range 0-3 for each question, with 0 representing low agreement, and 3 representing high agreement.

**Community stigma / Pèspektiv kominote a sou tibèkiloz / Perspectivas comunitarias hacia la tuberculosis**	Strongly disagree<br>*Fòtman pa dakò*<br>*Muy en Desacuerdo*	Disagree<br>*Pa dakò*<br>*En Desacuerdo*	Agree<br>*Dakò*<br>*De Acuerdo*	Strongly agree<br>*Fòtman dakò*<br>*Muy de Acuerdo*
A1. Some people may not want to eat or drink with friends who have TB<br>*Gen kèk moun ki ka pa vle manje oswa bwè ak zanmi ki gen TB*<br>*A algunas personas no les gusta beber or comer con amigos/as que padecen TB*	0	1	2	3
A2. Some people feel uncomfortable about being near those with TB<br>*Gen kèk moun ki santi yo pa alèz paske yo toupre moun ki gen TB*<br>*Algunas personas se sienten incómodas al estar cerca de quienes padecen TB*	0	1	2	3
A3. If a person has TB, some community members will behave differently towards that person for the rest of his/her life<br>*Si yon moun gen TB, kèk manm kominote a ap konpòte yon lòt jan anvè moun sa a pandan tout rès lavi li*<br>*Si alguien tuviera TB, algunas personas de la comunidad se comportarían diferentes con él/ella por el resto de su vida*	0	1	2	3
A4. Some people do not want those with TB playing with their children<br>*Gen moun ki pa vle moun ki gen TB jwe ak pitit yo*<br>*Algunas personas no quieren que sus hijos jueguen con personas que tienen TB*	0	1	2	3
A5. Some people keep their distance from people with TB<br>*Gen kèk moun ki kenbe distans yo ak moun ki gen TB*<br>*Algunas personas mantienen su distancia de aquellas con TB*	0	1	2	3
A6. Some people think that those with TB are disgusting<br>*Gen moun ki panse moun ki gen TB yo degoutan*<br>*Algunas personas piensan que quienes padecen TB son desagradables o indeseables*	0	1	2	3
A7. Some people do not want to talk to others with TB<br>*Gen kèk moun ki pa vle pale ak lòt moun ki gen TB*<br>*Algunas personas no quieren hablar con quienes tienen TB*	0	1	2	3
A8. Some people are afraid of those with TB<br>*Gen kèk moun ki pè moun ki gen TB*<br>*Algunas personas le tienen miedo a quienes tienen TB*	0	1	2	3

A9. Some people try not to touch others with TB<br>*Gen kèk moun ki eseye pa manyen lòt moun ki gen TB*<br>*Algunas personas tratan de no tocar a otras con TB*	0	1	2	3
A10. Some people may not want to eat or drink with relatives who have TB<br>*Gen kèk moun ki ka pa vle manje oswa bwè ak fanmi ki gen TB*<br>*Algunas personas no quieren beber o comer con familiares que tienen TB*	0	1	2	3
A11. Some people prefer not to have those with TB living in their community<br>*Gen kèk moun ki prefere pa gen moun ki gen TB k ap viv nan kominote yo*<br>*Algunas personas prefieren que quienes padecen TB no vivan en su comunidad*	0	1	2	3
**Individual stigma / Pèspektiv pasyan yo sou tibèkiloz / Perspectivas de los pacientes hacia la tuberculosis**				
B1. Some people who have TB feel hurt because of how others react when they find out they have TB<br>*Gen kèk moun ki gen TB ki santi yo blese akoz fason lòt moun reyaji lè yo konnen yo gen TB*<br>*Algunas personas con TB se sienten lastimadas por la manera en que los demás reacciónan cuando se enteran que tienen TB*	0	1	2	3
B2. Some people who have TB lose friends when they share with them they have TB<br>*Gen kèk moun ki gen TB ki pèdi zanmi lè yo pataje avèk yo ke yo gen TB*<br>*Algunas personas con TB pierden a sus amigos/as cuando les comentan que tienen TB*	0	1	2	3
B3. Some people who have TB feel alone<br>*Gen kèk moun ki gen TB ki santi yo poukont yo*<br>*Algunas personas con TB se sienten solas*	0	1	2	3
B4. Some people who have TB keep their distance from others to avoid spreading TB germs<br>*Gen kèk moun ki gen TB ki kenbe distans yo ak lòt moun pou evite gaye jèm TB yo*<br>*Algunas personas con TB guardan su distancia de otras para evitar contagiarlas con gérmenes de TB*	0	1	2	3
B5. Some people who have TB are afraid to tell those outside their family that they have TB<br>*Gen kèk moun ki gen TB ki pè di moun ki pa fanmi yo ke yo gen TB*<br>*Algunas personas con TB tienen miedo de decirles a otros aparte de su familia que tienen TB*	0	1	2	3
B6. Some people who have TB are afraid of going to TB clinics because other people may see them there<br>*Gen kèk moun ki gen TB ki pè ale nan klinik pou TB paske lòt moun ka wè yo la*<br>*Algunas personas con TB tienen miedo de ir a las clínicas de TB porque otras podrian ver*	0	1	2	3

B7. Some people who have TB are afraid to tell others that they have TB because others may think that they also have AIDS<br>*Gen kèk moun ki gen TB ki pè di lòt moun ke yo gen TB paske moun sa yo ka panse yo gen SIDA*<br>*Algunas personas con TB tienen miedo de decirles a otros que tiene TB pues pueden pensar que también tienen SIDA*	0	1	2	3
B8. Some people who have TB feel guilty because their family has the burden of caring for them<br>*Gen kèk moun ki gen TB ki santi yo koupab paske fanmi yo gen yon chay pou pran swen yo*<br>*Algunas personas con TB se sienten culpables por ser una carga para su familia*	0	1	2	3
B9. Some people who have TB will choose carefully who they tell about having TB<br>*Gen kèk moun ki gen TB k ap chwazi ak anpil atansyon ki moun yo ka di ke yo gen TB*<br>*Algunas personas que tienen TB son cuidadosas al escoger a quien decirle que padecen TB*	0	1	2	3
B10. Some people who have TB feel guilty for getting TB because of their smoking, drinking, or other careless behaviors<br>*Gen kèk moun ki gen TB ki santi yo koupab paske yo gen TB akoz ke yo fimen, bwè, oswa gen lòt konpòtman neglijan.*<br>*Algunas personas con TB se sienten culpables de haber contraído TB por fumar, tomar o por otras conductas irresponsables*	0	1	2	3
B11. Some people who have TB are worried about having AIDS<br>*Gen kèk moun ki gen TB ki gen enkyetid pou yo ta gen SIDA tou*<br>*Algunas personas con TB están preocupadas porque podrían tener SIDA*	0	1	2	3
B12. Some people who have TB are afraid to tell their family that they have TB<br>*Gen kèk moun ki gen TB ki pè di fanmi yo ke yo gen TB*<br>*Algunas personas con TB tienen miedo de decirle a sus familiares que tienen TB*	0	1	2	3
